# Supplementary material for: Effects of Milk Replacer-Based Lactobacillus on Growth and Gut Development of Yaks’ Calves: a Gut Microbiome and Metabolic Study
Source: Microbiol Spectr. 2022 Jun 30;10(4):e01155-22. doi: 10.1128/spectrum.01155-22 (PMC9431445; doi:10.1128/spectrum.01155-22)
Supplement: Supplemental file 1 — Supplemental material. Download spectrum.01155-22-s0001.pdf, PDF file, 0.1 MB [file spectrum.01155-22-s0001.pdf]

Table S1 The information of partly significantly different gut metabolits in the ESI+ and ESI- mode between FC and FW groups (VIP > 1 and P < 0.05).

Metabolits	FC_Mean	FW_Mean	Fold_change	P value	VIP	Ion mode
4,4-dimethyl-5alpha-chol est-7-en-3beta-ol	0.00025	0.00012	0.48575	0.032	1.364	ESI+
zymosterol	0.00189	0.00088	0.46566	0.007	1.693	ESI+
Ascorbic acid	0.00002	0.00003	1.62427	0.016	1.476	ESI-
Orotidylic acid	0.00006	0.00010	1.63429	0.010	1.554	ESI-
Cytosine	0.00003	0.00000	0.05598	0.016	1.576	ESI-
Orotic acid	0.00007	0.00011	1.66357	0.017	1.503	ESI-
Dihydrouracil	0.00080	0.00003	0.03509	0.018	1.595	ESI-

Table S2 The information of partly significantly different gut metabolits in the ESI+ and ESI- mode between FM and FW groups (VIP > 1 and P < 0.05).

Metabolits	FM_Mean	FW_Mean	Fold_change	P value	VIP	Ion mode
L-Isoleucine	0.00021	0.00053	2.53289	0.0004	1.774	ESI+
Nicotinic acid	0.00132	0.00270	2.03540	0.0495	1.217	ESI+
L-Proline	0.00040	0.00079	1.96866	0.0000	1.942	ESI+
L-Tyrosine	0.00098	0.00218	2.23634	0.0015	1.664	ESI+
Piperidine	0.00011	0.00026	2.45674	0.0004	1.755	ESI+
Novobiocin	0.00000	0.00000	5.72905	0.0338	1.322	ESI+
Thiamine	0.00008	0.00013	1.63128	0.0201	1.346	ESI+
Riboflavin	0.00040	0.00054	1.34150	0.0416	1.385	ESI+
Biotin	0.00066	0.00109	1.66358	0.0372	1.308	ESI+
Phylloquinone	0.00438	0.01014	2.31428	0.0459	1.206	ESI+
Stearidonic Acid	0.00012	0.00025	1.99514	0.0195	1.349	ESI+
alpha-Linolenic acid	0.00005	0.00015	2.86328	0.0178	1.337	ESI+
Sodium deoxycholate	0.09175	0.25058	2.73116	0.0075	1.933	ESI-
L-Phenylalanine	0.00080	0.00238	2.95969	0.0039	1.859	ESI-
Ursodeoxycholic acid	0.00001	0.00003	5.41500	0.0309	1.653	ESI-
Lithocholic acid	0.03325	0.11916	3.58406	0.0120	1.709	ESI-
L-Tyrosine	0.00069	0.00262	3.82206	0.0132	1.731	ESI-
Chenodeoxycholate	0.00447	0.01808	4.04917	0.0061	1.846	ESI-
Scopoletin	0.00002	0.00006	3.18919	0.0003	2.099	ESI-

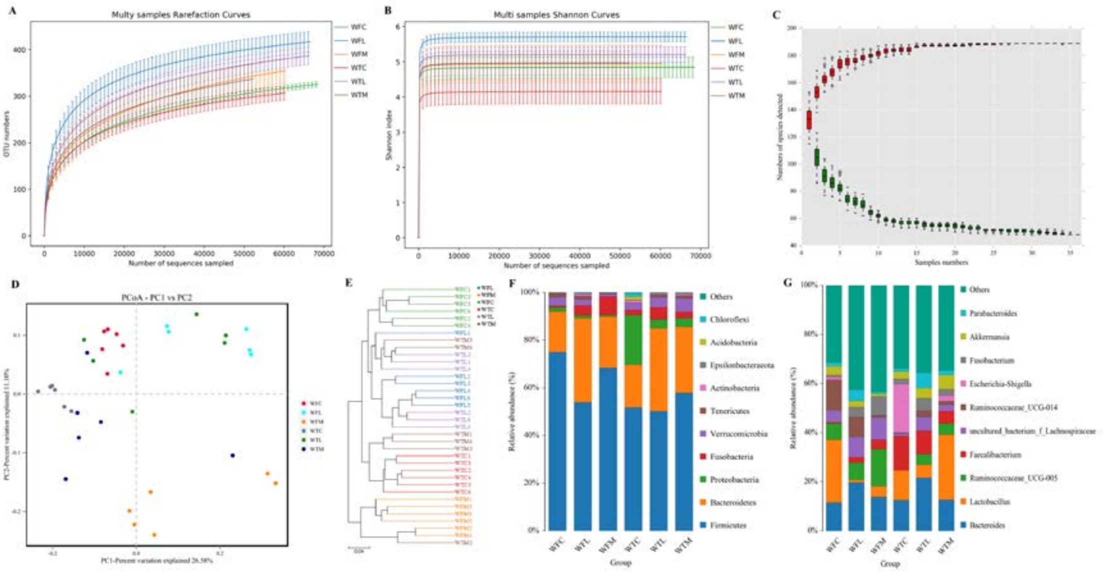

Supplementary Fig. 1 Effects of LAB-MR supplementation on gut bacterial communities' structures. A, Rarefaction curve; B, Rank abundance curve; C, Species accumulate curve; D, E represent the microbial similarity between groups by using PCoA scatter plot and UPGMA, respectively; F, G represent the analysis of microbial communities' structures at the phylum and genus levels, respectively.

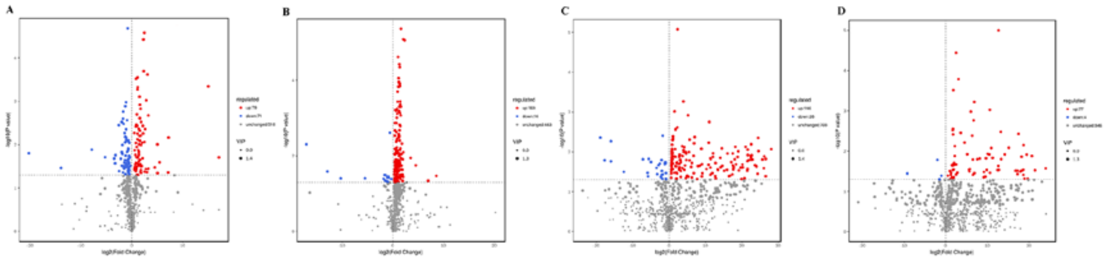

Supplementary Fig. 2 Volcano map showed the metabolites that were significantly up- or down-abundant driven by LAB-MR supplement in the ESI+ (A, B) and ES- modes (C, D). A, C represented the comparison between the FC group and the FW group; B, D represented the comparison between the FM group and FW group.
